# Prevalence and factors associated with intimate partner violence among the adolescent girls and young women in South Africa: findings the 2017 population based cross-sectional survey

**DOI:** 10.1186/s12889-021-11183-z

**Published:** 2021-06-16

**Authors:** Jacqueline Mthembu, Musawenkosi Mabaso, Sarah Reis, Khangelani Zuma, Nompumelelo Zungu

**Affiliations:** 1 Human and Social Capabilities Division, Human Sciences Research Council, Pretoria, South Africa; 2 University of California, Los Angeles, USA; 3 Human and Social Capabilities Division, Human Sciences Research Council, Durban, South Africa; 4 United Nations Population Fund, Pretoria, South Africa; 5 School of Public Health, University of Witwatersrand, Johannesburg, South Africa; 6 Department of Psychology, University of Pretoria, Pretoria, South Africa

**Keywords:** Intimate partner violence, Adolescent girls and young women, South Africa

## Abstract

**Background:**

Evidence indicate that intimate partner violence (IPV) is disturbingly high among South African adolescent girls and young women (AGYW). Understanding prevalence and risk factors for IPV among these emerging adults is critical for developing appropriate interventions to prevent adverse health outcomes later in life. This study investigates the prevalence and factors associated with lifetime physical IPV experience among AGYW, aged 15–24 years, using the South African national HIV prevalence, incidence, behaviour and communication survey conducted in 2017.

**Methods:**

The data used in this secondary analysis was obtained from a cross-sectional, population-based household survey data, conducted using a multi-stage stratified random cluster sampling approach. Multivariate stepwise backward logistic regression modelling was used to determine factors associated with IPV.

**Results:**

Of 716 AGYW that responded to the two commonly answered questions on IPV, 13.1% (95% CI: 9.6–17.6) indicated that they experienced IPV. The odds of reporting experiences of IPV were significantly lower among AGYW residing in high SES households [AOR = 0.09 (95% CI: 0.02–0.47), *p* = 0.004] than low SES households, and those residing in rural informal/tribal areas [AOR = 0.01 (95% CI: 0.00–0.22), p = 0.004] than urban areas. AGYW experiencing IPV had higher odds of reporting psychological distress compared to their counterparts [AOR = 4.37 (95% CI, 0.97–19.72), *p* = 0.054].

**Conclusion:**

The findings highlight the need for targeted structural and psychosocial interventions in low SES households and especially in urban areas.

## Background

Intimate partner violence (IPV) experienced by women is a global public health concern. IPV refers to any act of physical and sexual aggression or harm, sexual coercion, controlling behaviours, psychological/emotional abuse within an intimate relationship by a current or former partner/spouse [1]. Despite numerous intervention efforts over the last decade, the burden of IPV continues to grow [2]. The World Health Organization report global IPV estimates of just over 30% among ever-partnered women with similar rates estimated across sub-Saharan Africa [3]. South Africa is among the countries with the highest rates of IPV experienced by women in the world [4]. A growing body of research show that IPV among adolescent girls and young women (AGYW) is receiving increased attention due to its widespread nature and severe health consequences [5].

The negative health consequences of IPV (childhood/lifetime/past year experience) include adverse physical health outcomes (injury and death), poor mental health (depression and anxiety), sexual health risks (sexually transmitted infections including HIV), and reproductive health risks (unwanted pregnancy and abortion) [6–9]. IPV also leads to negative social consequences such as substance abuse which include alcohol misuse and drug use [9]. To inform IPV prevention efforts it is important to measure and understand factors associated with experiences of IPV.

Current evidence shows that factors associated with IPV (childhood/lifetime/past year experience) include low educational status, low socio-economic status, and gender inequalities including substance misuse by either the woman or her partner [10, 11]. The foremost psychological consequences of IPV include depression, post-traumatic stress disorder, and substance use disorders. Women with histories of IPV have been shown to suffer subsyndromal symptoms of mental health disorders, such as psychological distress symptoms [9–11]. Conversely, others have shown that women who experienced psychological distress were at increased risk IPV [9, 11]. This has been attributed to neurocognitive impairments that interfere with ability to evaluate danger, social skill and problem-solving deficits, stigma, and social isolation [9]. Other factors associated with IPV include sexual risk factors such as age disparate sexual relationships, transactional sex, and having multiple sexual partners [11]. IPV has also been associated with unequal gender power dynamics in relationships emanating from gender inequitable and harmful masculinities [7, 9, 12–15].

The South African Government in response to the growing scourge of IPV has pledged to address violence against women its causes and consequences through collection of relevant data in order to inform prevention activities [16]. Despite these progressive reforms, under-reporting remains a challenge and the Government’s response remains sporadic and unsatisfactory. Improved understanding of the burden of IPV and associated factor risk is vital to achieve a more complete picture of the true burden and its effect within populations in order to inform ongoing prevention efforts. However, little evidence exists on population-based prevalence and risk factors for IPV in South Africa. The aim of this paper was to investigate the prevalence and factors associated with lifetime physical IPV experience among the AGYW using a population-based household survey conducted in 2017 in South Africa.

## Methods

### Data source

The data used in this secondary analysis was obtained from the South African national HIV prevalence, incidence, behaviour and communication survey conducted in 2017. This is a cross-sectional, population-based household survey conducted using a multi-stage stratified random cluster sampling approach [17]. Basically, a total of 1000 small area layers (SALs) were sampled using the 2015 national population sampling frame of 103,000 SALs developed by Statistics South Africa [4]. The selection of SALs was stratified by province, locality type (urban formal, rural formal, and rural informal/tribal areas) and race groups in urban areas. A total of 15 visiting points (VPs) were randomly selected from each of the 1000 SALs, targeting 15,000 VPs. Of these, 12,435 (82.9%) VPs were approached. Among these VPs, 11,776 (94.7%) were valid and household response rate of 82.2% was achieved from the valid VPs. All members of selected households were eligible to participate in the survey.

Survey instruments included a household questionnaire and three age-appropriate questionnaires administered to consenting individuals. Questionnaire data were collected digitally using electronic tablets. For those younger than 18 years of age, consent was given by parents/guardians and assent by the participant. The interview instruments solicited information among others on demographic, behavioural, social and health characteristics of the sample. Data were weighted to account for the differential selection probabilities at the enumeration areas, households, and individual levels. The weights were benchmarked to the Statistics South Africa national midyear population estimates by age, race, sex and province to ensure that the data was nationally representative. The focus of the present paper was on AGYW 15–24 years who responded to the questions on experiences of IPV, and only female data for this age group was extracted from the national dataset.

### Measures

#### Primary outcome variable

One adult member aged 15 years and older, from each household was randomly selected to participate in the IPV module deals with violence experienced in intimate relationships. Discretion and confidentiality were maintained during the survey to assure the privacy and safety of all respondents. Due to the sensitive nature of the subject responses to most of the questions were extremely low. Hence, the primary outcome measure lifetime physical IPV experience was based only on the commonly answered questions “Did your partner ever do any of the following things to you that could hurt you?

- Question 1: Push you, shake you, or throw something at you? (Yes = 1 and No = 2).
- Question 2: Slap you? (yes = 1 and no = 2).

The responses were coded and dichotomised to generate IPV as follows:

- IPV (No = 0) if question 1 = 2 and question 2 = 2.
- IPV (Yes = 1) if question 2 = 1 and question 2 = 1.

#### Explanatory variables

Explanatory measured included socio-demographic characteristics such as age (15–19 years and 20–24 years), race (Black Africans and other race groups, which included Whites,

Coloureds, and Indians/Asians), marital status (married and not married; which included divorced/separated and widowed/widow), educational level completed (no education, primary, secondary, and tertiary), employment status (not employed and employed), locality type (urban formal, rural informal, rural formal), and asset-based socio-economic status (SES) which was constructed using multiple correspondence analyses (MCA) based on questions on the availability of essential services and ownership of a range of household assets [18]. MCA is a data reduction technique for categorical data, which calculates a composite indicator score computed by adding up all the weighted responses. The predicted score for each household was used to compute five quintiles (1st lowest, 2nd lower, 3rd middle, 4th higher and 5th highest) representing a continuum of household SES from the poorest to the least poor. The quintiles were then dichotomized into low SES or poorest (lowest 3 quintiles) high SES or less-poor (highest 2 quintiles).

Other explanatory variables included behavioural, social and health related factors such as alcohol use was measured using the Alcohol Use Disorders Identification Test (AUDIT) risk score (0 = abstainers; 1–7 = low-risk drinkers; 8–19 = high-risk drinkers; 20+ = hazardous drinking) [19], which has been validated in South Africa [20], orphanhood (not orphan, orphan) and, Psychological distress was derived from the 10 item Kessler psychological distress scale (K10) [21]. The K10 scale appraises of items on how respondents felt during the previous 30 days on a 5-point Likert scale (1 = never, 2 = rarely, 3 = some of the time, 4 = most of the time, 5 = all of the time). Cronbach’s alpha (0.92) indicated a high level of internal consistency of the K10 scale. Raw scores were summed, and the scale was dichotomized into two categories with a total score ≤ 20 for absence of psychological distress and > 20 for presence of psychological distress.

### Statistical analysis

Descriptive statistics was used to summarize the characteristics of the study sample, and differences between categorical variables was assessed using a Pearson chi-square test. Multivariate stepwise logistic regression analysis using a backward procedure was used to identify factors associated with IPV among AGYW. Probability for removal of variables in the model were set at *p*-values of 0.20. Adjusted odds ratios (AORs) with 95% confidence intervals (CI) are reported, and p-values less than 0.05 were considered statistically significant. Coefficient plots were used to display the results of the final model. All statistical analysis was done in Stata version 15.0 software using *“svy”* commands to take into account the complex multilevel survey design (Stata Corporation, College Station, Texas, USA).

## Results

### Characteristics of the study sample

Table 1 describe the study sample. Most of the study participants were young women (20–24 years), Black African, never married, had secondary level education, and were unemployed. More than a half AGYW resided in low SES households. Most resided in urban areas, were abstainers, were not orphans, and reported absence of psychological distress.

**Table 1:** Demographic, behavioural, social and health related characteristics of the study sample, adolescent girls and young women (15–24 years), South Africa

Variables	N^a^	%
**Age group in years**		
15–19	224	26.4
20–24	492	73.6
**Race groups**		
African	594	87.9
other	122	12.1
**Marital status**		
Married	32	5.8
Never married	681	94.2
**Education level**		
No education/Primary	34	6.2
Secondary	356	80.2
Tertiary	38	13.6
**Employment status**		
Not employed	616	86.1
Employed	95	13.9
**Asset based SES**^b^		
Low SES	374	54.6
HIGH SES	286	45.4
**Locality type**		
Urban	392	64.6
Rural informal (tribal areas)	253	30.0
Rural (farms)	71	5.4
**AUDIT score**^c^		
Abstainers	477	73.1
Low risk drinkers (1–7)	101	19.6
High risk drinkers (8–19)	46	5.6
Hazardous drinkers (20+)	5	1.7
**Orphanhood**		
Not orphan	188	62.7
Orphan	112	37.3
**Psychological distress**		
Absent	576	77.9
Present	137	22.1

^a^Subtotals do not all equal to the total (N) due to non-response and / or missing data, ^b^socio-economic status, (SES), ^c^alcohol risk score based on a questionnaire for Alcohol Use Disorder Identification Test (AUDIT)

### Prevalence of IPV

Of 716 AGYW that responded to the two commonly answered questions on IPV, 13.1% (95% CI: 9.6-17.6) indicated that they experienced lifetime physical IPV.

**Fig. 1:**
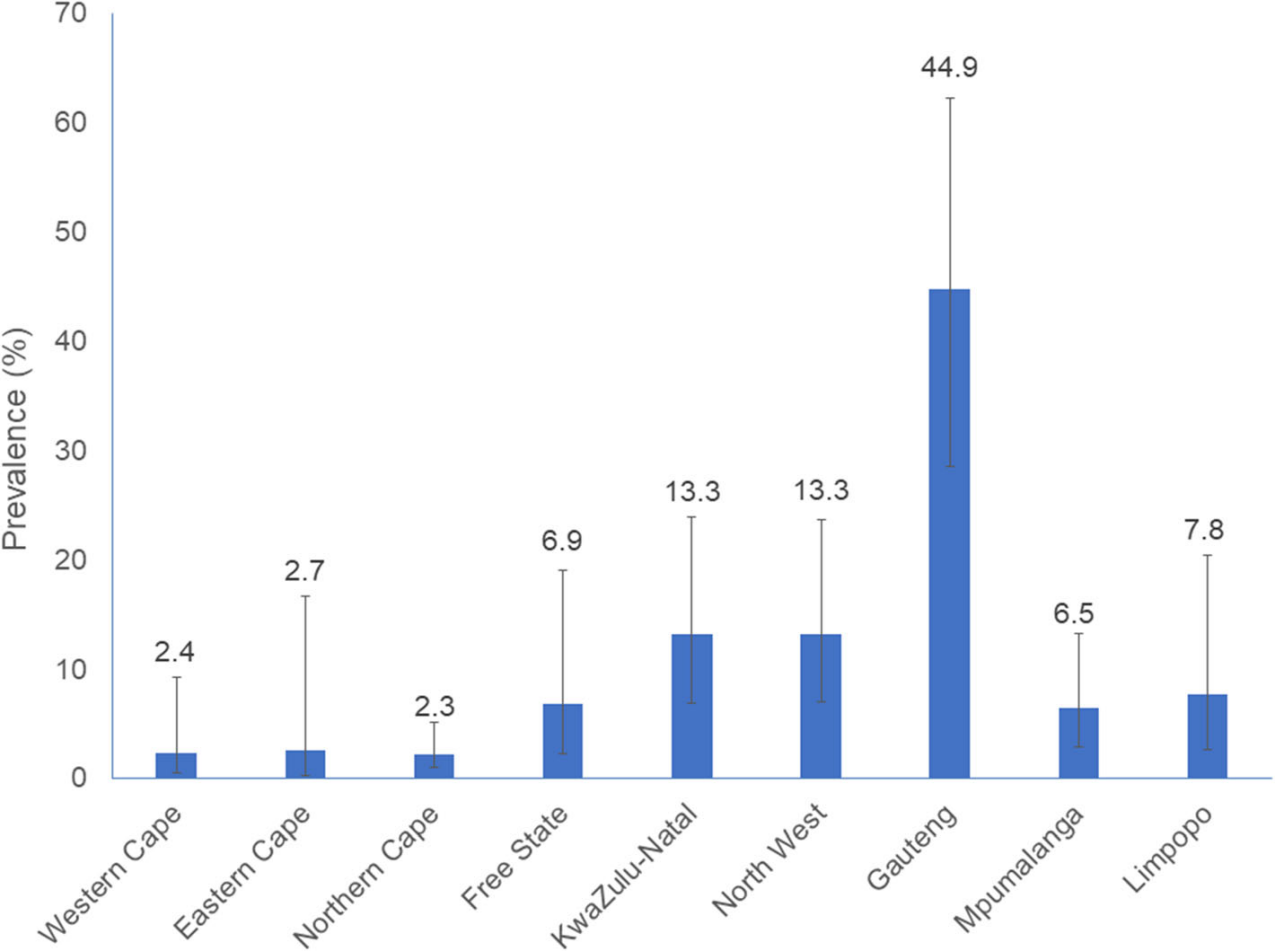
Prevalence of lifetime intimate partners violence experience by province, South Africa, 2017

Figure 1 shows that the prevalence of IPV was highest in the Gauteng province at 44.8% followed by KwaZulu-Natal and the Eastern Cape with prevalence at 13.3%, respectively.

**Table 2:** Prevalence of experience of lifetime physical IPV among adolescent girls and young women by demographic, behavioural, social and health related variables, South Africa 2017

Variables	Intimate partner violence (IPV)			
	N^a^	%	95% CI	p-value
**Age groups in years**				
15–19	224	14.2	6.9–27.1	0.766
20–24	492	12.7	9.1–17.4	–
**Race groups**				
African	594	13.8	9.9–19.0	0.091
other	122	7.7	4.1–14.0	–
**Marital status**				
Married	32	3.6	0.8–13.8	0.039
Never married	681	13.7	10.0–18.6	–
**Education level**				
No education/Primary	34	17.0	6.7–36.8	0.194
Secondary	356	17.8	12.5–24.5	–
Tertiary	38	4.9	0.8–25.8	–
**Employment status**				
Not employed	616	13.8	9.9–19.0	0.223
Employed	95	7.9	3.2–18.2	–
**Asset based SES**^b^				
Low SES	374	14.3	9.8–20.3	0.464
HIGH SES	286	10.9	5.7–19.9	–
**Locality type**				
Urban	392	14.0	9.2–20.7	0.580
Rural informal (tribal areas)	253	10.8	6.6–16.9	–
Rural (farm areas)	71	14.8	7.1–28.2	–
**AUDIT score**^c^				
abstainers	477	11.10	7.7–15.8	0.523
low risk drinkers (1–7)	101	15.2	7.9–27.2	–
high risk drinkers (8–19)	46	14.9	4.2–41.2	–
hazardous drinkers (20+)	5	31.4	5.2–79.2	–
**Orphanhood**				
not orphan	188	18.0	9.2–32.3	0.037
orphan	112	6.1	2.6–13.9	–
**Psychological distress**				
Absence	576	11.9	8.5–16.3	0.290
Presence	137	17.9	8.8–32.9	

^a^Subtotals do not all equal to the total (N) due to non-response and / or missing data, ^b^socio-economic status, (SES), ^c^alcohol risk score based on a questionnaire for Alcohol Use Disorder Identification Test (AUDIT)

Table 2 shows that the prevalence of lifetime physical IPV experience was significantly high among those not married, and those not orphaned. Even though not statistically significant, experiences of lifetime physical IPV was also higher among Black Africans, the unemployed, those from low SES households, and those with psychological distress.

### Factors associated with lifetime physical IPV among AGYW

**Fig. 2:**
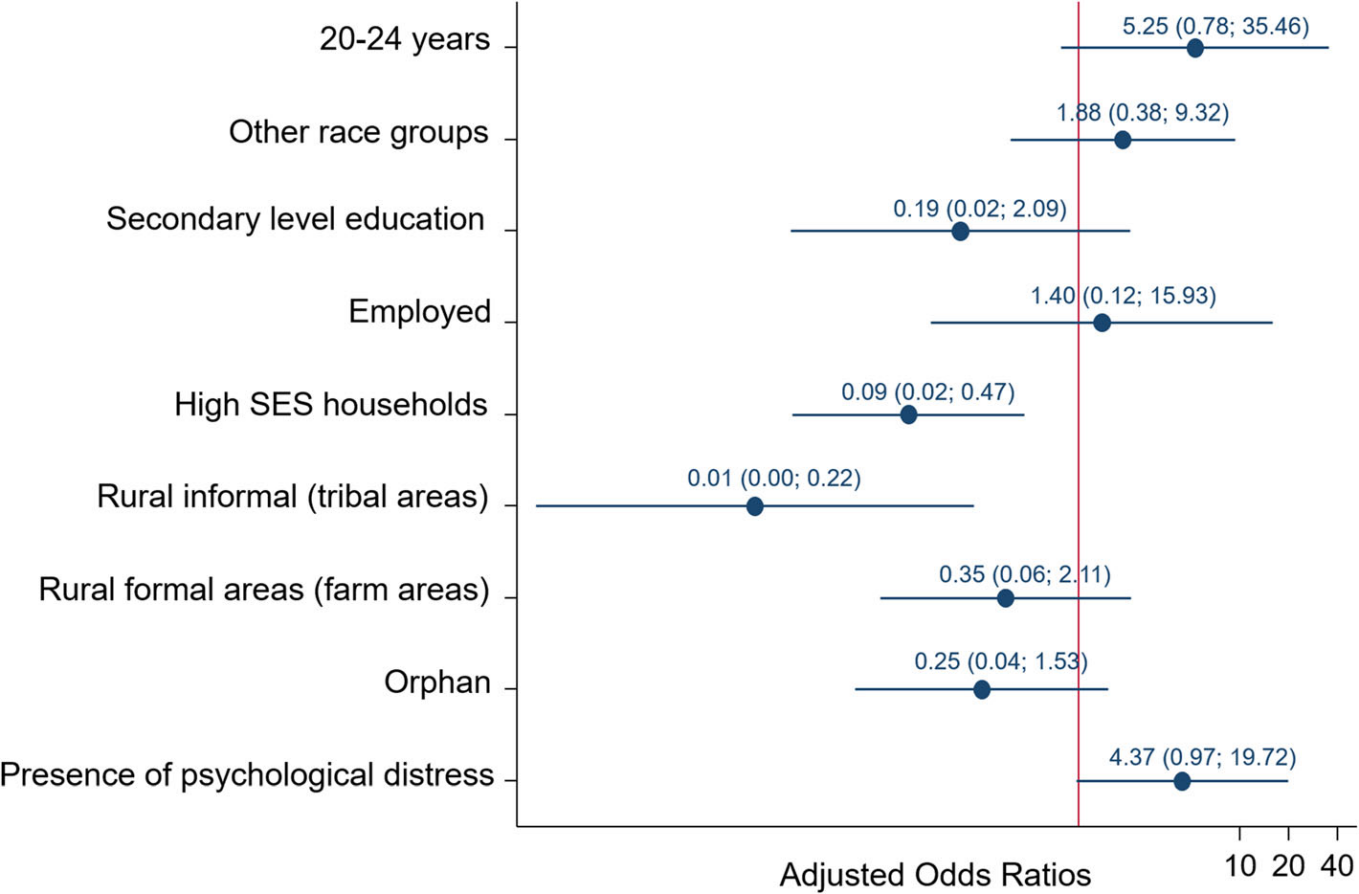
Multivariate model of factors associated with lifetime physical IPV experience among adolescents and young women, South Africa, 2017

Figure 2 presents the multivariable model of factors associated with lifetime physical IPV experience among AGYW 15–24 years. The odds of reporting experiences of lifetime physical IPV were significantly lower among AGYW residing in high SES households compared to those in low SES households [AOR = 0.09 (95% CI: 0.02–0.47), *p* = 0.004], and those residing in rural informal/tribal areas [AOR = 0.01 (95% CI: 0.00–0.22), p = 0.004] compared to urban areas. AGYW experiencing IPV had higher odds of reporting psychological distress compared to their counterparts [AOR = 4.37 (95% CI: 0.97–19.72), *p* = 0.054].

## Discussion

This is the first nationally representative cross-sectional survey focusing on prevalence and risk factors for lifetime physical IPV experience, among AGYW in South Africa. This secondary data analysis revealed that nationally the prevalence of lifetime physical IPV experiences among AGYW was 13.1%. This is lower than earlier provincial estimates reported in another study at 19.5 and 24.1% [4]. These low prevalence estimates could be due to the fact that 1) the national survey, unlike other IPV studies, focused on many other health related outcomes with a particular focus on HIV, and 2) only one household member was randomly selected to respond to IPV section of the questionnaire [17]. These observations highlight the need to prioritise IPV research especially among AGYW. The prevalence of lifetime physical IPV experience in this study varied with respect to a number of socio-demographic and socio-behavioural characteristics. Significant variations were between provinces with Gauteng reporting the highest IPV prevalence followed by KwaZulu-Natal and Eastern Cape provinces. These observations provide important clues for tailored and targeted approaches for improving IPV interventions.

In the final model lifetime physical IPV experiences was inversely associated with residing in high SES than low SES households. This probably reflects a cascade of interacting social vulnerabilities, which include limited access to social resources in low SES households which heightens exposure to IPV [22, 23]. In fact, evidence suggests that IPV can be concentrated in social-environments or settings with poverty and other dimensions of disadvantage [22, 23]. This implies that preventive measures and social support measures for IPV victims should be tailored for and targeted at social vulnerabilities and social environmental contexts that put young women at risk of IPV.

In the current paper lifetime physical IPV experiences was also inversely associated with residing in rural than urban settings. Incongruent with these findings studies have shown that disadvantaged urban settings can exacerbate underlying gender-based power disparities, with young women subject to intensive gender-based harassment and a pervasive threat of sexual and physical violence [24, 25]. Evidence suggests that adverse socio-economic conditions influenced by early adversity and social hardship may prompt violence perpetration by men seeking to reclaim power prompting discord that leads to violence [26]. However, more research is needed for improved understand of the underlying processes of social hardship, social gender norms, masculine identity, and power dynamics within relationships where IPV is prevalent.

Furthermore, lifetime physical IPV experiences was significantly associated with psychological distress. Other studies found that women experiencing physical IPV were likely to suffer from depression and anxiety indicative of psychological distress [27, 28]. Although the mechanisms underlying the relationship between IPV and psychological distress need to be better understood, it is possible that early trauma such as childhood/past exposure and/or social exposure IPV may be underlying causes and contribute to increased risk of psychological distress [29]. Posttraumatic stress disorder has been identified in women exposed to IPV along with comorbid symptoms such as depression, anxiety, suicidality and sleep disturbances [8, 30]. It has been suggested that harm reduction interventions in young women can be archived through universal education which incorporates relationship health that educate and enquire about abusive behaviours and IPV particularly in reproductive and adolescent health settings [31–33].. In addition, school-based educational efforts targeting the youth should include information about IPV early interventions appropriate to help prevent adverse mental health outcomes later in life [29].

### Limitations of the study

The current paper has several limitations. The paper relies on data that were self-reported and is therefore prone to recall and social desirability bias, which may lead to under reporting, especially when dealing with such a sensitive subject. The fact that few people responded to the question on IPV in such a large survey could have also introduced selection bias. Even though age group (15–19/20–24) is controlled for in adjusted models factors assessed are likely affected by the age of the AGYW, unfortunately, the small sample size would not allow for further stratification. The survey instrument on IPV was limited to a few items and did not utilise the highly recommended WHO five item scale on IPV since focus was mainly on HIV and sexual health. The study could also be limited by unmeasured important covariates such as childhood traumas, which are typically predictive of IPV experience. Including gender norms or male controlling behaviours again known risk factors for IPV experience that capture social aspects of gender inequalities. In addition, due to its cross-sectional nature, the study can only demonstrate an association and cannot infer any causality about IPV. The small sub-sample may have an impact on the precision of the estimates especially given the wide confidence intervals for some of the results. Furthermore, stepwise multiple regression include bias in parameter estimation, inconsistencies among model selection algorithms, and an inappropriate focus or reliance on a single best model. We discuss each of these issues with examples. Future, surveys with larger sample sizes would allow for more robust model fitting. Nevertheless, this paper was based on a nationally representative sample that can be used to draw inference about factors associated with lifetime physical IPV experiences among AGYW in the country.

## Conclusion

This paper presents the first nationwide secondary data analysis of lifetime physical IPV experiences among AGYW in South Africa. The findings highlight the need for targeted structural interventions in low SES households especially in urban areas. This suggests a need or social interventions to deal with powerful social and economic forces that encourage males to control female behaviour and trap women in abusive relationships. Women’s economic empowerment strategies have been proposed as means to reduce women’s risk of physical and sexual abuse [34]. This should be coupled with changing social and cultural norms that support violence against women, and challenging gender roles that grant men authority over women through awareness and advocacy campaigns. In addition, the findings suggest the urgency of psychosocial support for victims of IPV in the identified setting. Furthermore, the findings suggest the urgency of developing strategic parental-centred interventions that teach parents how to improve the safety of their young women, while simultaneously imparting skills that could facilitate a greater and lasting resilience among young IPV victims/survivors. Finally, there is a need for nationally representative survey specific on IPV and other forms gender-based violence in order to guide policy and design more nuanced and targeted IPV interventions in the country.

## Data Availability

The datasets used and/or analysed during the current study are available from the corresponding author on reasonable request.
